# Common genetic and environmental contributions to anxiety sensitivity, anxiety, and cognitive symptoms of eating disorders in adolescence

**DOI:** 10.1007/s10802-024-01273-y

**Published:** 2024-12-11

**Authors:** Rachel Smail-Crevier, Gerome Breen, Thalia C. Eley, Lance M. Rappaport

**Affiliations:** Department of Psychology, University of Windsor, Windsor, Ontario, Canada N9B 3P4; Institute of Psychiatry, Psychology & Neuroscience, Kings College London; Social Genetic and Developmental Psychiatry Centre, Memory Lane, London, England SE5; Department of Psychology, University of Windsor; Department of Psychology, University of Windsor, Windsor, Ontario, Canada N9B 3P4

**Keywords:** adolescent, anxiety sensitivity, anxiety, behavioral genetics, eating disorder symptoms

## Abstract

Anxiety sensitivity may be associated with both anxiety and eating disorder symptoms, which could contribute to the frequent comorbidity of both syndromes. This study examined the common (i.e., correlated) genetic and environmental contributions to anxiety sensitivity, cognitive symptoms eating disorder severity, and anxiety symptoms to understand their co-occurrence in adolescence. This study analyzed data from the Twins Early Development Study. When twins were 16 years old (N=5,111 pairs), they self-reported anxiety sensitivity via the Child Anxiety Sensitivity Index and cognitive symptoms of eating disorder severity via four items from the Eating Disorder Diagnostic Scale. Parents reported adolescent anxiety symptoms via the Anxiety Related Behaviour Questionnaire. Common genetic and non-shared environmental factors contributed to phenotypic correlations among cognitive symptoms of eating disorders. Genetic and nonshared- environmental influences contributed to anxiety sensitivity and a latent variable of cognitive symptoms of eating disorder severity. Genetic, shared-, and nonshared-environmental influences contributed to anxiety symptoms. Common genetic and nonshared environmental influences contributed to anxiety sensitivity and anxiety symptoms, as well as anxiety sensitivity and cognitive symptoms of eating disorder severity. However, there was no evidence of common genetic or environmental contributions to anxiety symptoms and cognitive symptoms of eating disorder severity. This study implicates anxiety sensitivity as a potential cognitive process associated with both anxiety symptoms and cognitive symptoms of eating disorders.

## Introduction

Eating disorder symptoms are prevalent among community samples of adolescents (Furnham et al., 2002; Zeiler et al., 2016). Adolescence, which is characterized by substantial neural changes (Giedd, 2015), is a period of heightened risk for the emergence of psychopathology. Accordingly, the prevalence of eating disorder symptoms increases across childhood into adolescence and remains prevalent across the lifespan (Runfola et al., 2013; McCabe & Ricciardelli, 2004). Numerous risk factors for eating disorder symptoms also emerge during the adolescent period and may contribute the increased prevalence of cognitive eating disorder symptoms during adolescence. For instance, factors such as increase in body mass index (Gowers & Shore, 2001), increased time spent with peers, and increased time spent on social media have been linked to elevated eating disorder symptoms (Gowers & Shore, 2001) and may be particularly relevant during adolescence. Cognitive symptoms of eating disorders (e.g., body dissatisfaction, weight concern) represent core, transdiagnostic symptoms of eating disorders. For example, recent evidence from network analyses suggest that cognitive symptoms, such as the overevaluation of weight and shape, body dissatisfaction, desire to be thin, and cognitive restraint, may be central symptoms to a broad range of eating disorders (i.e., anorexia, binge eating disorder, bulimia) (DuBois et al., 2017; Forrest et al., 2018; Monteleone & Cascino, 2021). Cognitive symptoms of eating disorders broadly increase one’s risk of both behavioural symptoms of eating disorders (e.g., binge eating) (Johnson & Wardle, 2005), as well as the development of more severe clinical eating disorders (see Keel & Forney, 2013, for review). Eating disorder symptoms, including cognitive symptoms, are also associated with impaired quality of life and psychosocial impairment, including low self-esteem and comorbid internalizing and externalizing psychopathology (Zeiler et al., 2016). Importantly, the prevalence of cognitive symptoms of eating disorders, as well as the severity of impairment due to the presence of eating disorder symptoms peak during adolescence (Tiggemann, 2004). Therefore, research efforts to identify biopsychosocial factors implicated in cognitive symptoms of eating disorders during the at-risk period posed by adolescence may be particularly relevant to reduce widespread impairment due to eating disorder symptoms.

Among community samples of adolescents, genetic factors contribute substantially to both eating and anxiety symptoms and disorders (Chen et al., 2015; Sawyers et al., 2019; Waszczuk et al., 2019). Genetic factors account for between 30% to 75% of the variance in eating disorders and related symptoms, whilst nonshared environmental influences account for the remaining variance (Klump et al., 2001; Rowe et al., 2002; Waszczuk et al., 2019). Genetic contributions increase, while shared environmental influences decrease across pubertal development from childhood to adolescence (Klump et al., 2007). Therefore, factors such as puberty likely contribute to the wide range in heritability estimates of eating disorder symptoms among adolescents. Similar to eating disorder symptoms, genetic contributions to anxiety symptoms and disorders range from 20% to 60% throughout childhood and adolescence (Eley et al., 2003; Hallett et al., 2009; Scaini et al., 2012). Environmental influences also contribute to child and adolescent liability to anxiety disorders and symptoms, though longitudinal research suggests that environmental influences may be disorder- and time-specific (Nivard et al., 2015; Patterson et al., 2018). Specifically, during early childhood shared environment, whilst during later childhood and adolescence nonshared environment, may contribute more substantially to anxiety symptoms and disorders (Eley et al., 2003; Hallett et al., 2009; Kendler et al., 2008; Lamb et al., 2010; Sawyers et al., 2019; Scaini et al., 2012). Thus, similar to eating disorders and symptoms, developmental status and syndrome assessed likely contribute to the wide range of heritability estimates found for anxiety symptoms and disorders (Patterson et al., 2018).

Anxiety symptoms and disorders are frequently comorbid with eating disorder symptoms among community samples of adolescents (Herpertz-Dahlmann et al., 2008; Touchette et al., 2011). For example, compared to those without any eating pathology, adolescent girls who presented with elevated eating disorder symptoms or a subclinical eating disorder were more likely to have symptoms of separation anxiety disorder and generalized anxiety disorder (Touchette et al., 2011). Empirical findings suggest that common genetic and environmental influences contribute to both anxiety and eating disorder symptoms (Dellava et al., 2011; Silberg & Bulik, 2005), suggesting that common factors or processes may be implicated in both anxiety and eating disorder symptoms. Anxiety-related cognitive processes, such as anxiety sensitivity, may be implicated in both anxiety and eating disorder symptoms and may partially explain their high concordance (Touchette et al., 2011; Zeiler et al., 2016). Specifically, anxiety sensitivity is a cognitive bias that may reflect common genetic and environmental contributions to both anxiety and eating disorder symptoms. Indeed, like anxiety and eating disorder symptoms, genetic and environmental factors contribute to anxiety sensitivity in adolescents. For instance, in adolescents aged 12 to 19 years, genetic influences accounted for 60% of the variance in anxiety sensitivity; with remaining variance being attributed to shared and person-specific environmental factors (Brown et al., 2012).

Anxiety sensitivity is conceptualized in extant literature as a heightened attention to, and fear of, anxiety, including anxiety-related cognitions and bodily sensations such that they are perceived as indicative of harm (Taylor, 1999). Theoretically, anxiety sensitivity may contribute to worsened anxiety symptoms (e.g., panic disorder) as the fear of anxiety-related symptoms increases attention to anxiety-related cues (e.g., related bodily sensations), as well as subsequent distress acutely or about anticipated experiences of anxiety or fear (e.g., about anticipated panic attacks; Reiss, 1987). In the context of eating disorder symptoms, anxiety sensitivity may be theoretically extended to include heightened sensitivity to cognitions and interoceptive sensations (i.e., bodily) related to eating and digestive cues (e.g., fullness). Thus, elevated anxiety sensitivity may increase i) one’s attention to eating and digestive cues; ii) the perceived association of eating and digestive cues with negative outcomes (e.g., weight gain); and iii) one’s subsequent tendency to interpret eating and digestive cues as indicative of harm. Anxiety sensitivity may also increase demand to regulate negative affect or distress, which may be experienced both more frequently and as more aversive. Consistent with theoretical foundations for disordered eating behaviours (Hearon et al., 2014), an individual might use disordered eating (e.g., binge eating) as an affect regulation strategy. If so, elevated anxiety sensitivity may increase both the frequency and severity of cues to employ disordered eating to downregulate distress.

Prior research implicates anxiety sensitivity in myriad psychiatric conditions including symptoms of anxiety (Noël & Francis, 2011) and eating disorders (Davey & Chapman, 2009; DeBoer et al., 2012), although results have been mixed when examining individual dimensions of anxiety sensitivity (i.e., social, physical, and cognitive) (Espel-Huynh et al., 2019). Moreover, preliminary findings demonstrate that anxiety sensitivity shares genetic and environmental correlations with both anxiety (Hettema et al., 2020) and eating disorder symptoms in adolescence (Fairweather-Schmidt & Wade, 2020). Specifically, Fairweather-Schmidt & Wade (2020) demonstrate common genetic and environmental contributions to both anxiety sensitivity and eating disorder symptoms broadly among female adolescents. The present study aims to extend these findings in three primary ways. First, the present study seeks to explore whether these findings generalize to a larger, mixed-sex sample. Second, the present study seeks to clarify the common (i.e., correlated) genetic and environmental influences to anxiety sensitivity and, specifically, cognitive symptoms of eating disorders given their high prevalence in adolescence (Furnham et al., 2002; Zeiler et al., 2016), as well as their implication in a broad range of eating disorders (Monteleone & Cascino, 2021). Finally, the present study included an assessment of concurrent anxiety symptoms to evaluate the degree to which common genetic and environmental contributions of anxiety sensitivity with both anxiety symptoms and cognitive symptoms eating disorder severity might explain the phenotypic correlation of anxiety symptoms with cognitive symptoms eating disorder severity (Herpertz-Dahlmann et al., 2008; Touchette et al., 2011).

### The present study

The present study sought to examine the genetic and environmental contributions to anxiety sensitivity, cognitive symptoms eating disorder severity, and anxiety symptoms to understand the role of common genetic and environmental influences in their co-occurrence in adolescence. In order to evaluate this overarching hypothesis, we first examined the genetic and environmental contributions to cognitive symptoms of eating disorders with the hypothesis that common genetic and environmental influences would contribute to the intercorrelation among eating disorder symptoms. We also hypothesized that genetic influences would contribute significantly to a latent factor that indexed cognitive symptoms eating disorder severity. Finally, in line with the overarching goal of the study, we hypothesized that genetic and environmental contributions to anxiety sensitivity, cognitive symptoms eating disorder severity, and anxiety symptoms would be intercorrelated, which would indicate common (i.e., correlated) genetic and environmental contributions to anxiety sensitivity, anxiety symptoms, and cognitive symptoms of eating disorders.

## Materials and Methods

### Participants

The present study used data collected between February 2011 and June 2012 when twins were approximately 16 years old. The mean age of participants was 16.3; participant age ranged from 14.9 to 19.4 years. At first contact, when twins were 18 months old, the sample included primarily white children (91.7%), female children (50.1%), employed mothers (43.1%), and employed fathers (91.7%), which was representative of the general population from which the sample was recruited (Rimfeld et al., 2019). Similar percentages remained throughout the study (see, Rimfeld et al., 2019, for more detailed demographic information for the TEDS sample). In total, 5,111 twin pairs provided data at this assessment wave, of which 55.3% of twins were female and 64.4% twin pairs were monozygotic. At this assessment wave, 10,868 pairs of twins were initially contacted, of which 93.5% were white. A total of 5,144 twin pairs provided data; 33 twin pairs were excluded because information on twin zygosity was unavailable. Twin zygosity was assessed by DNA test or a well-validated parent-report questionnaire, which shows 95% agreement with DNA assay of zygosity (Price et al., 2000). As a secondary analysis of data, the sample size was predetermined. However, the present sample provides 80% statistical power to identify small genetic contributions to a latent composite of cognitive symptoms eating disorder severity (e.g., a^2^ = 0.15; see data analysis below). The present sample also provides 80% statistical power to identify a moderate correlation among the genetic or environmental contributions to multiple phenotypes, such as anxiety sensitivity and latent cognitive symptoms eating disorder severity (e.g., *rA* =0.30) (Verhulst, 2017).

### Procedure and Ethical Considerations

The present study leveraged data from the Twins Early Development Study (TEDS), a longitudinal genetic epidemiological study of pairs of monozygotic and dizygotic twins born in England and Wales between 1994 and 1996 (Lockhart et al., 2023; Rimfeld et al., 2019). Twins were first recruited at 18 months of age; the most recent assessment wave occurred when twins were 21 years old. Twin births were identified through birth records and families were contacted. A total of 16,810 twin pairs initially indicated interest in participating in the study; at first contact, 13,694 pairs of twins provided data. The number of twin pairs enrolled in the study decreased slightly in early childhood (*n*=10,150), middle childhood (*n*=8,819), and adolescence (*n*=8,697). For the present adolescent sample, both parents and twins provided written consent prior to completing questionnaires. Previously disseminated research examined anxiety sensitivity and anxiety symptoms using data from this sample (Cheesman et al., 2018; Madrid-Valero et al., 2020; Peel et al., 2023; Singham et al., 2017; Waszczuk et al., 2015). No prior research examined eating disorder symptoms or its association with anxiety sensitivity or anxiety symptoms in the sample used for the present study. This secondary analysis of previously collected data was cleared by the University of Windsor Research Ethics Board.

### Measures

#### Cognitive Symptoms of Eating Disorders

Twins self-reported cognitive symptoms of eating disorders via four items from the Eating Disorder Diagnostic Scale (Stice et al., 2000), which measures eating disorder symptoms over the past 6 months. Specifically, four questions were used to assess feeling fat, fear of gaining weight, and the influence of weight and shape on self-evaluation. Each item was completed on a 7-point Likert scale from “not at all” (1) to “extremely” (7). Rather than compute a total score for cognitive symptoms of eating disorders based on the four items, cognitive symptoms eating disorder severity was assessed as a latent factor indexed by the four symptom items. Prior research demonstrates strong reliability and validity of the full Eating Disorder Diagnostic Scale including criterion validity, test-retest reliability, and internal consistency (Stice et al., 2000).

#### Anxiety Sensitivity

Twins completed the Child Anxiety Sensitivity Index (Peterson & Heilbronner, 1987), which is comprised of 18 self-reported items on a three-point Likert scale (i.e., none, some, a lot). Total anxiety sensitivity is the sum of all 18 items. Prior research indicates moderate to good internal consistency for each of the four factor scales, with alphas ranging from 0.62 to 0.80 among children and adolescents (Silverman et al., 2003). (Silverman et al., 2003). The present study identified similarly high internal consistency (α = 0.92) and evidence that the scale is unifactorial (ω_hierarchical_ = 0.64) particularly as a single higher-order factor of multiple subfactors (ω_total_ = 0.94).

#### Anxiety symptoms

Parents reported on anxiety over the past 6 months for each adolescent twin via the Anxiety Related Behaviour Questionnaire, which includes 19 items rated on a 3-point Likert scale (i.e., not true, quite true, very true). Total anxiety symptoms was computed as the sum of all 19 items. While prior research reports varying factor structures of the Anxiety Related Behaviour Questionnaire as a function of child age, high internal consistency for the full scale is stable across samples and ages assessed, which provides evidence for a consistent, reliable unifactorial structure (Eley et al., 2003; Hallett et al., 2009). The present study agrees with prior research to document high internal consistency (α = 0.92) and evidence that the scale may be unifactorial (ω_hierarchical_ = 0.70) particularly as a single higher-order factor of multiple subfactors (ω_total_ = 0.94).

### Data Analysis

Analyses were conducted using the biometrical statistical twin model in a structural equation modelling framework (Evans et al., 2002). The biometrical twin model leverages the difference in genetic concordance between monozygotic and dizygotic twins, who share 100% and 50% of their segregating genes, respectively. Thus, the variance in one trait (e.g., cognitive symptoms of eating disorders) or covariance between two traits (e.g., anxiety sensitivity and cognitive symptoms of eating disorders) can be decomposed into additive genetic (i.e., the combined contribution of more than one gene to a phenotype), shared environmental (i.e., influences of environment shared between twins within a family), and nonshared environmental (i.e., environmental influences specific to each child) influences (Neale & Cardon, 1992). First, we used the biometrical twin model to estimate genetic and environmental covariances among the four cognitive symptoms of eating disorders. Each of the four cognitive symptoms of eating disorders were regressed onto child age and biological sex (see Supplementary Table S1, available online). Participant sex, but not age, was significantly, strongly associated with each symptom (see Table 1). To adjust for potential associations with cognitive symptoms of eating disorders and other constructs under investigation, age and sex were included as covariates in subsequent analyses. Next, we estimated the additive genetic (A), shared environmental (C), and nonshared environmental (E) contributions to a latent composite of cognitive symptoms eating disorder severity indexed by the four cognitive symptoms of eating disorders assessed, and evaluated the potential existence of additional higher-order factors of cognitive symptoms of eating disorders (Kendler et al., 1992). The final analytic model then extended this model to simultaneously estimate the phenotypic correlation of latent cognitive symptoms eating disorder severity and other latent eating disorder factors with anxiety sensitivity and anxiety symptoms as well as estimate correlations among genetic, shared environmental, and nonshared environmental contributions to latent cognitive symptoms of eating disorder severity, anxiety sensitivity, and anxiety symptoms to explain their well-documented phenotypic intercorrelation.

Analyses were conducted in the open source R statistical framework (R Core Team, 2020), using the OpenMx package (Neale et al., 2016) to estimate structural equation models with full information maximum likelihood estimation via the NPSOL optimizer, and the psych package (Revelle, 2015) to estimate descriptive data (e.g., psychometric data). Cognitive symptoms of eating disorders were treated as ordinal to test assumptions underlying the biometrical twin model. For all other analyses (e.g., estimation of phenotypic, genetic, and environmental correlations), analysis of items as ordinal proved infeasible with the substantial computational resources available. Therefore, variables were treated as ordinal whenever possible and continuous when necessary. Analysis of ordinal data as continuous may overestimate the contribution of variance attributable to nonshared-environmental contributions (Verhulst & Neale, 2021). However, differences between ordinal and continuous correlations would be considered a small effect (i.e., *r*=0.10), especially as the number of ordinal categories increases (Verhulst & Neale, 2021).

## Results

### The structure of genetic and environmental contributions to cognitive symptoms of eating disorder severity

Preliminary analyses verified biometrical statistical model assumptions (Supplemental Table S2). Additive genetic and non-shared environmental factors contributed substantially to between-person variance in feeling fat, fear of gaining weight, and the influence of weight on self-evaluation. Shared and nonshared environmental factors contributed substantially to between-person variance in the symptom that one’s shape influences self-evaluation (Table 2). Moreover, as theoretically expected, all four cognitive symptoms of eating disorders were strongly correlated phenotypically (Table 2). Evident highly correlated genetic and nonshared environmental influences to all four cognitive symptoms of eating disorders suggest the presence of a latent cognitive symptom of eating disorder severity factor through which common genetic and nonshared environmental factors influence all individual cognitive symptoms of eating disorders (Table 2).

**Figure 1:**
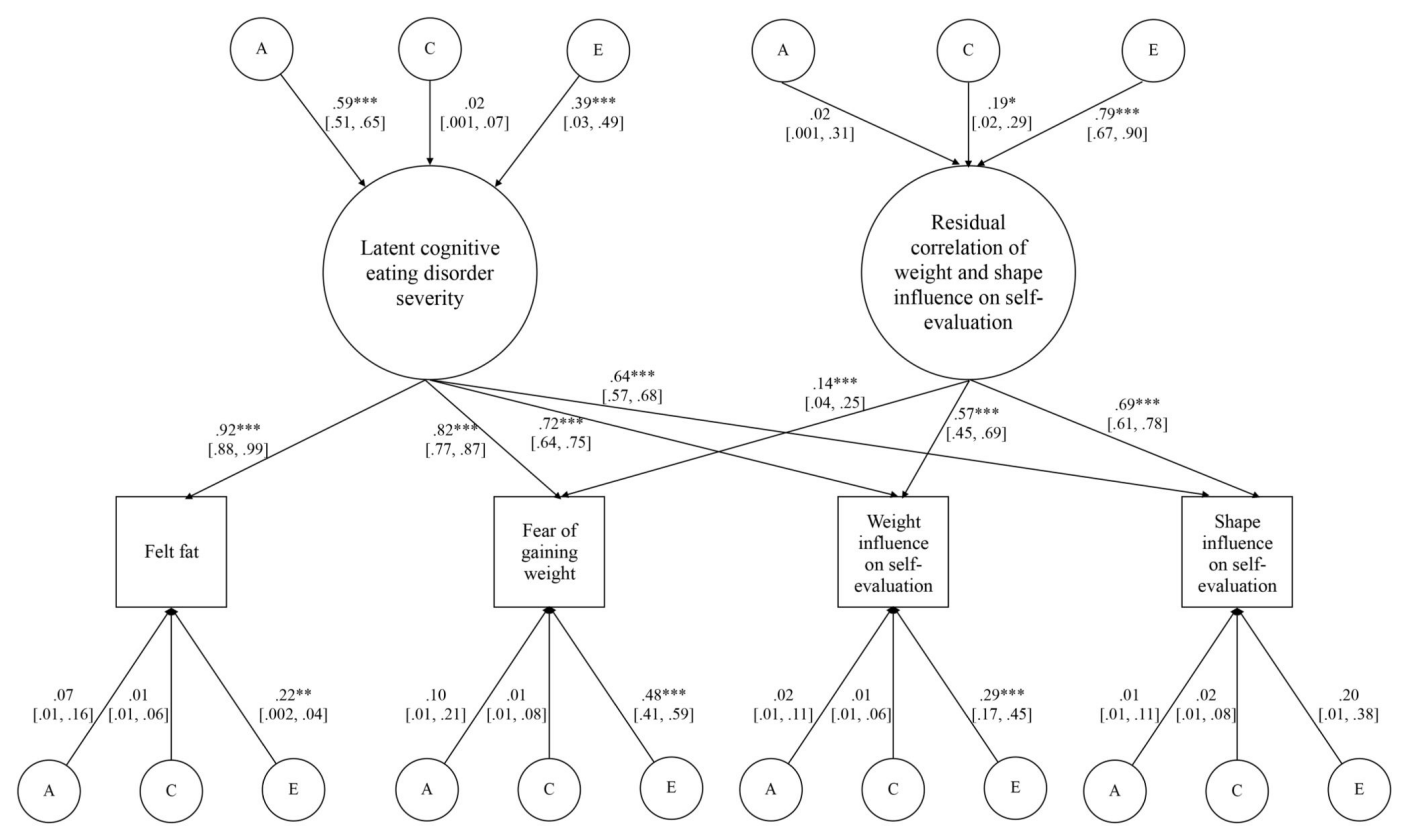
Genetic and environmental contributions to latent cognitive symptoms eating disorder severity *Note*. Additive genetic (A). Shared environment (C). Nonshared environment (E). *p<.05. **p< .01. ***p< .001

Therefore, we next fit a latent factor extension to the biometrical statistical model, which included two orthogonal latent factors to model latent cognitive symptoms eating disorder severity and given their high conceptual similarity, a possible residual correlation between the two items that index whether one’s weight or shape influences self-evaluation (see Figure 1). This model was nested within the original model, in which all four cognitive symptoms were intercorrelated. Hence, model fit for the latent two-factor model was assessed by comparison using the chi-square difference test and Akaike Information Criterion (AIC). The chi-square index of model fit indicates no statistically significant loss of fit in the two-factor model, χ^2^(8834)=30709.83, AIC=30783.83, as compared to the original correlated factor model, χ^2^(8827)=30700.11, AIC=30787.11, Δχ^2^(7)=9.73, *p*=.20. The two-factor model of cognitive symptoms of eating disorders also provided improved model fit as compared to a model with one latent cognitive symptoms of eating disorder severity factor, χ^2^(3)=700.25, *p*<.0001.

Additive genetic and nonshared environmental influences, but not shared environmental influences, contributed significantly to latent cognitive symptoms eating disorder severity. Shared and nonshared environmental influences, but not additive genetic influences, contributed significantly to variance in the residual correlation of weight and shape influence on self-evaluation. After accounting for both latent factors (i.e., cognitive symptoms eating disorder severity and residual correlation of weight and shape on self-evaluation), the residual variance of each cognitive symptom of eating disorders (e.g., fear of gaining weight) was partitioned into genetic, shared, and nonshared environmental influences. Only nonshared environmental influences contributed to residual variance in each cognitive symptom of eating disorders (see Figure 1).

### Phenotypic, genetic, and environmental correlations among anxiety sensitivity, eating disorder symptoms, and anxiety symptoms

Phenotypically, anxiety sensitivity, latent cognitive symptoms of eating disorder severity, the residual correlation of weight and shape influence on self-evaluation, and anxiety symptoms were moderately intercorrelated with the caveat that the two latent factors from cognitive symptoms of eating disorders were necessarily orthogonal factors (Table 3). Additive genetic and nonshared environmental influences contributed to between-person variance in anxiety sensitivity, latent cognitive symptoms of eating disorder severity, and anxiety symptoms. Shared environmental influences also contributed to variance in anxiety symptoms (Table 3).

As hypothesized, genetic contributions to anxiety sensitivity were significantly, moderately correlated with genetic contributions to both latent cognitive symptoms of eating disorder severity and anxiety symptoms. Nonshared environmental contributions to anxiety sensitivity were also significantly, modestly correlated with nonshared environmental contributions to latent cognitive symptoms of eating disorder severity and anxiety symptoms as well as the residual correlation of weight and shape influence on self-evaluation. Contrary to hypotheses, there was no evidence of common genetic or environmental contributions to latent cognitive symptoms of eating disorder severity and anxiety symptoms (Table 3). There were also no evident shared environmental correlations among anxiety sensitivity, anxiety symptoms, latent cognitive symptoms of eating disorder severity, and the residual correlation of weight and shape influence on self-evaluation. This is likely due to low contributions of shared environmental influences to these phenotypes overall.

## Discussion

Consistent with previous research and shared liability models of psychopathology (Klump et al., 2010; Waszczuk et al., 2019), the present findings suggest that common genetic influences are partially responsible for the coherence of cognitive symptoms of eating disorders. Nonshared environmental influences contributed to both the coherence and variability in the presentation of cognitive symptoms of eating disorders. For example, this study provides evidence that a set of environmental influences, including both shared (i.e., familial) and nonshared environmental influences, uniquely contributed to the influence of weight and shape on one’s self-evaluation. This extends prior evidence that self-evaluation of weight and shape may be particularly influenced by environmental factors (Reichborn-Kjennerud et al., 2004; Wade & Bulik, 2007). For example, numerous nonshared environmental influences, including social media use (Wade et al., 2017) and perceived pressure to be thin (Wilksch & Wade, 2010) may contribute to the elevated influence of weight and shape on self-evaluation. While more research is needed on the shared (i.e., familial) environmental contributions to the influence of weight and shape on self-evaluation, prior research documents potential influences of familial factors such as parent concern about a child’s weight (Field et al., 2001) and father’s sensitivity to reward (Wilksch & Wade, 2010). The present findings emphasize both shared and nonshared environmental contributions to the influence of weight and shape on self-evaluation, which represents a core, transdiagnostic symptom of eating disorders in adolescence. However, further research is needed to clarify the influence of familial factors on the undue influence of weight and shape concern on self-evaluation.

Consistent with prior research, genetic and nonshared environmental influences contributed to anxiety sensitivity (Zavos et al., 2012) and anxiety symptoms (Nivard et al., 2015). The present findings indicate that familial influences may continue to contribute broadly to anxiety symptoms in middle adolescence, which extends prior research in community samples of adolescents (Eley & Stevenson, 1999; Scaini et al., 2012) although evidence is mixed (Nivard et al., 2015). Multiple factors, such as age at assessment and the anxiety syndrome assessed may contribute to inconsistent estimates in prior research (Patterson et al., 2018).

Further, as hypothesized based on theory and prior research, anxiety sensitivity, anxiety symptoms, and cognitive symptoms of eating disorder severity were phenotypically correlated with one another (Fairweather-Schmidt & Wade, 2020; Qi et al., 2021; Touchette et al., 2011). This research extends prior research among youth (Zavos et al., 2012) to suggest that, in adolescence, common genetic and nonshared environmental contributions to anxiety sensitivity and anxiety symptoms may contribute to their phenotypic correlation.

Moreover, the present findings similarly suggest common genetic and nonshared environmental influences to anxiety sensitivity and cognitive symptoms of eating disorder severity may contribute to their phenotypic correlation. This evidence is consistent with a growing body of evidence that implicates anxiety sensitivity in adolescent psychopathology beyond anxiety syndromes (Noël & Francis, 2011; Qi et al., 2021) including in eating disorder symptoms (Anestis et al., 2007; Davey & Chapman, 2009). These present findings notably build upon one study that identified common genetic and environmental contributions to anxiety sensitivity and eating disorder symptom severity broadly (i.e., both behavioural and cognitive symptoms of eating disorders) in a community sample of specifically female adolescents (Fairweather-Schmidt & Wade, 2020). The present study extends the findings from this previous study by leveraging a large epidemiological sample and including both female and male participants, which demonstrates the generalizability of the previous findings to a larger, mixed sex sample. This development addresses a longstanding need for research on eating disorder symptoms to include male participants, especially given the high and increasing prevalence of eating disorder symptoms in this population (Zeiler et al., 2016). The present results also demonstrate that anxiety sensitivity shares common genetic and environmental influences specifically with cognitive symptoms of eating disorder severity, which may represent a distinct construct from disordered eating behaviours (e.g., Christian et al., 2020).

Anxiety sensitivity is broadly conceptualized as enhanced attention to, and fear of, cognitions and body-related cues associated with anxiety (Taylor, 1999). In light of the present findings, anxiety sensitivity may capture the multifaceted construct of interoceptive processes, which encompass heightened sensitivity to bodily sensations, as well as negative beliefs, thoughts, and worry about one’s bodily sensations beyond those associated to anxiety (see Garfinkel, Seth, Barrett, Suzuki, & Critchley, 2015, for review). From this perspective, a heightened attention to, and fear of, body-related cues associated with weight, shape, and eating concern (e.g., stomach distension) may increase the frequency and intensity with which one negatively experiences their own body, leading to overall maladaptive thoughts related to their body, weight, and shape. Previous research suggests that elevated attention to and awareness of internal physiological cues may contribute to body dissatisfaction in community samples of women and men (Bekker et al., 2002), and that heightened interoceptive awareness may mediate the association of anxiety sensitivity with eating disorder symptoms (Anestis et al., 2007). However, further research is required to explicate the mechanisms through which anxiety sensitivity is associated with cognitive symptoms of eating disorders. Cognitive symptoms of eating disorders represent core, transdiagnostic symptoms of eating disorders such that they are prevalent among a wide range of eating disorders including anorexia, bulimia, and binge eating disorder (DuBois et al., 2017; Monteleone & Cascino, 2021). Moreover, cognitive symptoms, such as body dissatisfaction and weight and shape concerns have been implicated in the development of clinically relevant eating disorders (Keel & Forney, 2013). Therefore, given the current findings, anxiety sensitivity may represent a treatment target for a broad range of eating disorders and symptoms. Overall, the present results emphasize the broad contribution of anxiety sensitivity to psychopathology during adolescence and suggest that it may be an intervention target for both anxiety and eating disorder symptoms. Given the growing body of research that implicates anxiety sensitivity in eating disorder symptoms, further research is needed to evaluate the impact of targeting anxiety sensitivity for the treatment of eating disorders and symptoms.

The present findings should be considered in light of several limitations. First, anxiety symptoms were measured via parent report while anxiety sensitivity and cognitive symptoms of eating disorders were both measured via adolescent self-report. Parent-reported and adolescent-reported anxiety symptoms are modestly correlated (Muris et al., 2003). Therefore, the present results may be considered a lower bound estimate of the phenotypic correlation of anxiety symptoms with anxiety sensitivity or cognitive symptoms of eating disorder severity and an upper bound estimate of the phenotypic correlation of anxiety sensitivity with cognitive symptoms of eating disorder severity. Importantly, the present assessment method would not necessarily lead to higher concordance between monozygotic and dizygotic twins in the biometrical statistical models used. For example, the present study documents statistically significant genetic and environmental correlations between anxiety sensitivity and anxiety symptoms despite the assessment of anxiety symptoms via parent-report. Second, possible qualitative and quantitative effects of biological sex on the genetic and environmental contributions to cognitive symptoms of eating disorder severity were not assessed. While eating disorder symptoms may be somewhat more prevalent or severe among female adolescents, symptom etiology, course, and outcomes do not necessarily differ as a function of participant biological sex or gender (Baker et al., 2009; Reichborn-Kjennerud et al., 2004). Third, this study sought to assess the concurrent genetic and environmental associations among anxiety sensitivity, anxiety symptoms, and eating disorder symptoms in adolescence. Thus, the study used cross-sectional data of twins aged 16 years. Based on current theory that implicates anxiety sensitivity as an underlying cognitive bias that contributes to the development of psychopathology (Noël & Francis, 2011; Qi et al., 2021), we interpret the present results to suggest that anxiety sensitivity may increase liability to both anxiety symptoms and cognitive symptoms of eating disorders. However, longitudinal research is needed to further test this theoretical model. Finally, while the relative contribution of genetic versus environmental contributions to eating disorders and symptoms is generally stable across time and cohorts (Crisp et al., 1985; Waszczuk et al., 2019; Yao et al., 2021), sociocultural changes may correspond to varying shared and nonshared environmental influences on eating disorder symptoms. Indeed, research demonstrates that environmental contributions to eating disorder symptoms change across early childhood to adolescence (Klump et al., 2010; Waszczuk et al., 2019), conceivably, in part, due to changes in environment (e.g., spending less time with parents and more time with peers). For this reason, future research is needed to evaluate whether the present findings generalize to more current cohorts of adolescents recruited recently.

## Conclusion

Overall, we found that genetic and nonshared environmental influences contributed to a latent variable of cognitive symptoms of eating disorders, which represents a range of eating-and weight-related cognitions in adolescence. Beyond overall cognitive symptoms of eating disorder severity, a unique set of shared and nonshared environmental influences contributed specifically to the undue influence of weight and shape on self-evaluation. Thus, future research may benefit from examining environmental risk factors for the undue influence of weight and shape on self-evaluation. Phenotypically, anxiety sensitivity, anxiety symptoms, and cognitive symptoms eating disorder severity were intercorrelated. Common genetic and nonshared environmental influences contributed to shared liability to anxiety sensitivity and anxiety symptoms, as well as to shared lability to anxiety sensitivity and latent cognitive symptoms eating disorder severity. However, contrary to study hypotheses, common genetic and nonshared influences did not explain the phenotypic correlation of anxiety symptoms with cognitive symptoms eating disorder severity. These findings suggest that anxiety sensitivity is independently correlated with cognitive symptoms of eating disorder severity and anxiety symptoms. The present study accords with recent research to suggest a broad association of anxiety sensitivity with psychopathology including cognitive symptoms eating disorders, which represent transdiagnostic symptoms of eating disorders. However, future research is needed to explicate the mechanisms through which anxiety sensitivity may be associated with cognitive symptoms of eating disorders in adolescence.

## Supplementary Material

Supplementary Material

## Figures and Tables

**Table 1:** Descriptive statistics as a function of adolescent biological sex – Mean (SD)

Construct/Item	Total	Female	Male
Eating disorder cognitive symptoms			
Felt fat	2.29 (2.14)	3.06 (2.07)	1.12 (1.65)
Fear of gaining weight	2.14 (2.20)	2.92 (2.21)	0.94 (1.57)
Weight influence on self-evaluation	2.04 (2.19)	2.62 (2.25)	1.16 (1.77)
Shape influence on self-evaluation	2.12 (2.12)	2.59 (2.18)	1.39 (1.81)
Anxiety sensitivity	7.93 (5.98)	9.47 (6.32)	5.99 (4.87)
Anxiety symptoms	3.60 (4.22)	4.20 (4.48)	2.84 (3.73)

**Table 2:** Phenotypic, additive genetic, shared environmental, and nonshared environmental contributions to and correlations among eating disorder symptoms – Estimate [95% CI]

Phenotypic				
Symptom	Felt fat	Fear weight	Weight self	Shape self
Felt fat				
Fear weight	.75*** [.73, .77]			
Weight self	.66*** [.63, .68]	.67*** [.65, .69]		
Shape self	.59*** [.56, .62]	.62*** [.60, .65]	.86*** [.84, .87]	
Additive Genetic				
Symptom	Felt fat	Fear weight	Weight self	Shape self
Felt fat	.56*** **[.48, .61]**			
Fear weight	.83*** [.72, .94]	.47*** **[0.34, 0.53]**		
Weight self	.94*** [.76, 1.25]	.94*** [.75, 1.22]	.33*** **[0.14, 0.47]**	
Shape self	1.06*** [.72, 326.22]	1.00*** [.67, 303.39]	.99** [.76, 213.77]	**.16** **[0, .36]**
Shared Environment				
Symptom	Felt fat	Fear weight	Weight self	Shape self
Felt fat	**0 [0, .05]**			
Fear weight	8168.53 [-7.00x10^4^, 7.17x10^4^]	**0 [0, .10]**		
Weight self	19.73 [-1.21x10^5^, 8.15x10^4^]	22.44 [-1.22x10^5^, 9.91x10^4^]	**.08** **[0, .22]**	
Shape self	62.05 [-1.01x10^5^, 189.76]	93.63 [-1.09x10^5^, 220.44]	1.08* [.75, 180.83]	.19** **[.04, .33]**
Non-Shared Environment				
Symptom	Felt fat	Fear weight	Weight self	Shape self
Felt fat	.44*** **[.39, .50]**			
Fear weight	.63*** [.58, .69]	.53*** **[.47, .60]**		
Weight self	.48*** [.40, .54]	.52*** [.45, .58]	.60*** [.53, .67]	
Shape self	.42*** [.34, .50]	.48*** [.41, .55]	.80*** [.76, .83]	.65*** **[.57, .73]**

*Note*. Fear weight indicates fear of gaining weight; Weight self indicates the influence of weight on one’s self-evaluation; Shape self indicates the influence of shape on one’s self-evaluation. Additive genetic, shared environmental, and nonshared environmental contributions to each symptom are provided on the diagonal and bolded.

* p<.05.

** p<.01.

*** p<.001.

**Table 3:** Additive genetic, shared environmental, and nonshared environmental contributions to and correlations among anxiety sensitivity, latent cognitive symptoms eating disorder severity, and anxiety symptoms – Estimate [95% CI]

Phenotypic				
Syndrome	Anx Sens	Cog Eating	Eating Res	Anx Sev
Anx Sens				
Cog Eating	.22^***^ [.21, .23]			
Eating Res	.24^***^ [.23, .25]	0		
Anx Sev	.21^***^ [.20, .21]	.10^***^ [.09, .10]	.10^***^ [.09, .11]	
Additive Genetic				
Syndrome	Anx Sens	Cog Eating	Eating Res	Anx Sev
Anx Sens	.40^***^ [.40, .40]			
Cog Eating	.26^**^ [.21, .36]	.61^***^ [.60, .60]		
Eating Res	.91 [.86, .98]	0	**.02 [.02, .02]**	
Anx Sev	.37^***^ [.37, .40]	.06 [.01, .06]	.65 [.54, .87]	.39^***^ [.38, .39]
Shared Environmental				
Syndrome	Anx Sens	Cog Eating	Eating Res	Anx Sev
Anx Sens	**.002 [.002, .002]**			
Cog Eating	.24 [.19, .27]	**.005 [.005, .006]**		
Eating Res	.97 [.92, .98]	0	**.19 [.18, .20]**	
Anx Sev	.32 [.33, .33]	1.00 [.99, 1.00]	.08 [-.03, .17]	.27^***^ [.27, .27]
Non-Shared Environmental				
Syndrome	Anx Sens	Cog Eating	Eating Res	Anx Sev
Anx Sens	**.60 [.59, .60] ^a^**			
Cog Eating	.20^***^ [.21, .23]	**.39 [.39, .41] ^a^**		
Eating Res	.20 ^***^ [.19, .21]	0	**.79 [.80, .80] ^a^**	
Anx Sev	.11 ^***^ [.12, .12]	.09 [.09, .10]	.05 [.04, .06]	**.35 [.35, .35] ^a^**

*Note*. Anx Sens indicates anxiety sensitivity; Cog Eating indicates latent cognitive symptoms eating disorder severity; Eating Res indicates the latent residual correlation of weight and shape influence on one’s self-evaluation; Anx Sev indicates anxiety symptoms. Additive genetic, shared environmental, and nonshared environmental contributions to each construct are provided on the diagonal and bolded.

a We note that, since they include other person-specific influences (e.g., measurement error), the statistical significance of nonshared environmental contributions (E) to each phenotype cannot be directly estimated. We note that, in rare cases, confidence intervals do not include the associated point estimate due to numerical rounding and limited computational precision in confidence interval estimation through profile maximum likelihood estimation.

* p<.05.

** p<.01.

*** p<.001.

## Data Availability

The data associated with this research paper are available upon request from the Twins Early Developmental Study (TEDS; https://www.teds.ac.uk/researchers/teds-data-access-policy).
