# Supplementary material for: Common genetic and environmental contributions to anxiety sensitivity, anxiety, and cognitive symptoms of eating disorders in adolescence
Source: Res Child Adolesc Psychopathol. Author manuscript; Available in PMC 2025 Dec 18. (PMC7618485; doi:10.1007/s10802-024-01273-y)
Supplement: Supplementary Material [file EMS201834-supplement-Supplementary_Material.docx]

**Common etiology of anxiety sensitivity, anxiety, and cognitive eating disorder symptoms among adolescents**

Submitted to

Research on Child and Adolescent Psychopathology

**Supplemental Tables**

**Table S1. Cognitive eating disorder symptoms regressed on adolescent age and biological sex**

| Item | Age | | Sex | |
| --- | --- | --- | --- | --- |
|  | *b* | *p* | *b* | *p* |
| Felt fat | -.26 [-.67, .15] | .22 | -1.97 [-2.14, -1.81] | 1.72 x 10^-105^ |
| Fear weight | -.08 [-.50, .34] | .70 | -1.96 [-2.13, -1.79] | 2.14 x 10^-97^ |
| Weight self | -.06 [-.49, .37] | .78 | -1.43 [-1.62, -1.25] | 9.19 x 10^-51^ |
| Shape self | .04 [-.39, .47] | .85 | -1.17 [-1.36, -.99] | 9.77 x 10^-36^ |

## *Note.* Fear weight indicates the symptom Fear of gaining weight; Weight self indicates the symptom Weight influence on self-evaluation; Shape self indicates the symptom Shape influence on self-evaluation. Sex is coded such that 0 is female and 1 is male. These models were estimated using SLSQP optimization.

**Table S2. Evaluation of assumptions in statistical genetics: Effects of twin order and zygosity on thresholds for cognitive eating disorder symptoms**

| Felt fat | | | | | | |
| --- | --- | --- | --- | --- | --- | --- |
| Model | χ^2^ | df | AIC | Δ χ^2^ | Δ df | p |
| Fully saturated | 7967.54 | 2204 | 8019.54 |  |  |  |
| Item Thresholds Constrained Across Twin Order | 7977.36 | 2216 | 8005.36 | 9.81 | 12 | .63 |
| Item Thresholds Constrained Across Zygosity | 7983.18 | 2222 | 7999.18 | 15.63 | 18 | .62 |
|  |  | | | | | |
| Fear of gaining weight | | | | | | |
| Model | χ^2^ | df | AIC | Δ χ^2^ | Δ df | p |
| Fully saturated | 7663.15 | 2205 | 7715.14 |  |  |  |
| Item Thresholds Constrained Across Twin Order | 7668.58 | 2217 | 7696.58 | 5.43 | 12 | .94 |
| Item Thresholds Constrained Across Zygosity | 7671.31 | 2223 | 7687.31 | 8.16 | 18 | .98 |
|  |  | | | | | |
| Weight influence on self-evaluation | | | | | | |
| Model | χ^2^ | df | AIC | Δ χ^2^ | Δ df | p |
| Fully saturated | 7539.40 | 2204 | 7591.40 |  |  |  |
| Item Thresholds Constrained Across Twin Order | 7550.59 | 2216 | 7578.59 | 11.19 | 12 | .51 |
| Item Thresholds Constrained Across Zygosity | 7555.02 | 2222 | 7571.02 | 15.62 | 18 | .62 |
|  | | | | | | |
| Shape influence on self-evaluation | | | | | | |
| Model | χ^2^ | df | AIC | Δ χ^2^ | Δ df | p |
| Fully saturated | 7888.64 | 2200 | 7940.64 |  |  |  |
| Item Thresholds Constrained Across Twin Order | 7903.96 | 2212 | 7931.96 | 15.32 | 12 | .22 |
| Item Thresholds Constrained Across Zygosity | 7913.55 | 2218 | 7929.55 | 24.91 | 18 | .13 |

Note. These models were estimated using SLSQP optimization.
